# Ultrahigh verapamil-loaded controlled release polymeric beads using superamphiphobic substrate: D-optimal statistical design, *in vitro* and *in vivo* performance

**DOI:** 10.1080/10717544.2018.1482974

**Published:** 2018-06-14

**Authors:** Carol Yousry, Maha M. Amin, Ahmed H. Elshafeey, Omaima N. El Gazayerly

**Affiliations:** Department of Pharmaceutics and Industrial Pharmacy, Faculty of Pharmacy, Cairo University, Cairo, Egypt

**Keywords:** Verapamil hydrochloride, superamphiphobic substrates, encapsulation efficiency, D-optimal statistical design, *in vivo* pharmacokinetic study

## Abstract

Controlled-release multiparticulate systems of hydrophilic drugs usually suffer from poor encapsulation and rapid-release rate. In the present study, ultra-high loaded controlled release polymeric beads containing verapamil hydrochloride (VP) as hydrophilic model drug were efficiently prepared using superamphiphobic substrates aiming to improve patient compliance by reducing dosing frequency. Superamphiphobic substrates were fabricated using clean aluminum sheets etched with ammonia solution and were treated with 1.5% (w/v) perfluorodecyltriethoxysilane (PFDTS) alcoholic solution. The effect of the main polymer type (lactide/glycolide (PLGA) 5004A, PLGA 5010, and polycaprolactone (PCL)), copolymer (Eudragit RS100) content together with the effect of drug load on encapsulation efficiency (EE%) and *in vitro* drug release was statistically studied and optimized via D-optimal statistical design. *In vivo* pharmacokinetic study was carried out to compare the optimized system relative to the market product (Isoptin^®^). Results revealed that superamphiphobic substrates were successfully prepared showing a rough micro-sized hierarchical structured surface upon observing with scanning electron microscope and were confirmed by high contact angles of 151.60 ± 2.42 and 142.80°±05.23° for water and olive oil, respectively. The fabricated VP-loaded beads showed extremely high encapsulation efficiency exceeding 92.31% w/w. All the prepared systems exhibited a controlled release behavior with Q12 h ranging between 5.46 and 95.90%w/w. The optimized VP-loaded system composed of 150 mg (1.5% w/v) PCL without Eudragit RS100 together with 160 mg VP showed 2.7-folds mean residence time compared to the market product allowing once daily administration instead of three times per day.

## Introduction

The sustainability of multiple-dose a day encounters the challenge of patient compliance especially for those who suffer from chronic diseases. Thus, controlled release drug delivery formulations are considered as the appropriate choice allowing reduced dosing frequency with fewer side effects and comparable therapeutic efficiency relative to conventional formulation. It also provides reduced plasma level fluctuations with a possibility of dose reduction (Katz et al. 1995). Among controlled release delivery systems, multiparticulate systems offer extra advantages over single unit ones as they allow uniform drug absorption due to their uniform drug distribution all over the gastrointestinal tract (GIT). They also offer reduced inter and intra-subject variability, reduced local irritation as well as low risk of dose dumping that is highly associated with controlled release single unit dosage form (Dey et al., 2008; Rajabi-Siahboomi, 2017).

However; formulation of successful multiparticulate controlled release systems is limited by the physicochemical characteristics of the drug. Conventional microencapsulation techniques usually involve harsh conditions of temperature, pH, and stirring as well as the use of two or more liquid phases which compromise the drug stability and encapsulation within the particulate systems (Jain, 2000; Lima et al., 2012). Specifically, hydrophilic drugs are highly subjected to poor encapsulation within the multiparticulate systems as they diffuse to the external aqueous phase during preparation leaving most of the formulated particulate systems free of drug or with poor drug encapsulation (Jain, 2000). Additionally, those hydrophilic drug-loaded multiparticulate systems show relatively rapid drug release due to their rapid solubilization and diffusion of the drug to the external dissolution media (Nounou et al., 2006).

Researchers worked hard to overcome such formulation challenges. They turned to replace the external aqueous phase with less desirable one either by changing its pH, saturating it with an external electrolyte (Mobarak et al., 2014), or by changing the whole external phase (Yousry et al., 2017). Also, the drug release could be modulated by the selective choice of polymers or polymers combinations that strictly control drug release (Rajabi-Siahboomi, 2017).

Recent studies tend to use superhydrophobic surfaces to formulate highly drug encapsulated spherical multiparticulate drug delivery systems by excluding the external liquid phase. Hundred percent drug encapsulation is claimed as the whole process takes place at the solid-air interface without the interference of any other liquid thus, drug migration to the external doesn’t occur (Song et al., 2010; Lima et al., 2011; Puga et al., 2013). The main concern about such technique is the restriction offered by the solvent type, as only pure water can be used as solvent. This hinders its application with hydrophobic polymers which could be of special interest in controlled release formulations (Rial-Hermida et al., 2014).

Instead, superamphiphobic surfaces that show high contact angles and low wettability for both water and organic oils (Manna et al., 2017) could be a more promising universal method in drug formulations as it doesn’t offer any restrictions on the polymer type or the solvent used. Although several methods to fabricate superamphiphobic surfaces on different substrates were reported (Deng et al., 2012, 2017; Qing et al., 2017), only Rial-Hermida et al. (2014) applied such surfaces in pharmaceutical formulations. They managed to prepare superamphiphobic surface using glass substrates to be used in the formulation of ciprofloxacin- loaded lactide/glycolide (PLGA) particles for sustained release formulation.

Verapamil hydrochloride (VP) is one of the widely used calcium channel blocker in the pharmaceutical market (Yassin et al., 2006). It is effectively used as an antiarrhythmic agent to control tachyarrhythmias. Its potent vasodilating and negative ionotropic effect supports myocardial infarction and cardiomyopathy treatment (Choi & Burm, 2005).

Clinical studies have shown that oral administration of VP is one of the well-tolerated first line treatments for patients with essential hypertension as it is not associated with the common side effects such as orthostatic hypotension, reflex tachycardia, and tolerance present with other treatments (Jankowski et al., 1992). Upon oral administration, 90% of the ingested dose is absorbed from the GIT; however, only 20–30% of the dose is bioavailable in blood as it’s extensively metabolized in the liver. It has a relatively short half-life (4h) so it is given in a dose of 80 mg three times daily which hinders patient compliance (McTavish & Sorkin, 1989; Jankowski et al., 1992).

Controlled release multiparticulate systems of VP could be a promising approach to overcome such problem of compliance especially in patients with chronic diseases. Many studies performed to encapsulate VP into controlled release multiparticulate systems using different types of hydrophilic and hydrophobic polymers (Kiliçarslan & Baykara 2003; Yassin et al., 2006; Akmal et al., 2015; Jawed et al; 2017) failed to achieve high encapsulation due to its high water solubility and subsequently, its diffusion outside the fabricated systems leaving most of the particulates free from drug. Therefore, the formulation of controlled release multiparticulate systems with ultrahigh VP encapsulation would propose the desired approach.

In this study, we worked to evaluate the suitability of superamphiphobic substrates applications to highly encapsulate VP as a model hydrophilic drug into sustained release multiparticulate systems using hydrophobic polymers aiming to enhance patient compliance. Although previous studies dealt with superhydrophobic and superamphiphobic substrates in drug formulation assumed complete encapsulation of the drug (100%) inside the beads due to the exclusion of the external liquid phase without measuring, this study is the first to measure and calculate the encapsulation efficiency (EE%) by tracking any drug diffusion to the external superamphiphobic substrate surface. D-optimal statistical design was adopted to study the effect of main polymer type (polycaprolactone (PCL), PLGA 5004A, and (PLGA 5010), Eudragit RS100 content as a co-polymer, and the effect of drug load on the formulation conditions to achieve systems with highest EE% and controlled release behavior. Finally, *in vivo* study was performed on the optimized system compared to the market product Isoptin^®^ to assure its *in vivo* pharmacokinetic performance.

## Materials

Aluminum sheets (50 mm × 50 mm) and verapamil hydrochloride were supplied as a gift from El-Nasr Chemical Co. (Cairo, Egypt). PURASORB PDLG (DL-lactide/Glycolide copolymer) 5004 A (PLGA 5004A; composed of 50:50 lactide: glycolide molar ratio with an inherent viscosity of 0.4 dl/g) and PURASORB PDLG (DL-lactide/Glycolide copolymer) 5010 (PLGA 5010 composed of 50:50 lactide: glycolide molar ratio with an inherent viscosity of 1 dl/g) were supplied as a gift from Purac Biomaterials (Netherlands). PCL; average molecular weight (M. Wt.) 70,000–9,0000) was purchased from Sigma Aldrich Inc. Al. (St. Louis, Mo). Eudragit RS100 was supplied as a gift from Degussa, Rohm GmbH & Co. (Germany). 1H, 1H, 2H, 2H- perfluorodecyltriethoxysilane (PFDTS) was purchased from Finetech Industry limited (China). Ethyl alcohol (95%), hydrochloric acid, methylene dichloride, ammonia solution (33%), olive oil, and tribasic sodium phosphate were purchased from El Nasr pharmaceutical chemicals company (Cairo, Egypt).

## Methodology

### Preparation and characterization of superamphiphobic substrate

Superamphiphobic substrates were prepared by etching the surface of aluminum plates and formation of a rough low energy surfaces instead. Briefly, aluminum plates were ultrasonically cleaned with ethanol then distilled water each for 15 min. The cleaned plates were then immersed in 0.3 M ammonia solution for 4 h at 80 °C (heater water bath; Buchi B465; Buchi labortechnik AG, Switzerland) in a sealed container to etch the surface. Finally, the etched surface was washed with distilled water and immersed in 1.5% (w/v) 1H, 1H, 2H, 2H- perfluorodecyltriethoxysilane (PFDTS) alcoholic solution for 48 h then left in air for drying (Peng & Deng, 2015).

The roughness of the substrate was observed by scanning electron microscope (SEM) (Quanta 250 FEG; FEI company, Netherlands) after spraying with gold (Emitech K550X sputter coater; Quorum Technologies, England). The static contact angles (CA) of water, olive oil, and later, the optimized selected system solution from the designed study were measured as follows:

A 5 µl liquid droplet was allowed to drop on surface of the treated aluminum plates and the CA was measured using contact angle meter (DM-701; KYOWA Interface Science co., Ltd., Japan) interfaced by KYOWA interface measurement and Analysis system FAMAS software version 3.4. The same measurements were carried out on untreated aluminum plates for comparative purposes. The recorded results are the mean values of three sample measurements (Peng & Deng, 2015).

### Preparation of VP-loaded polymeric beads

A three-factor D-optimal design was applied to evaluate and statistically optimize the factors affecting the preparation of controlled release VP-loaded polymeric beads. The studied independent variables were set to one categoric factor (main polymer type) and two other numeric factors namely Eudragit RS100 content (percentage out of the total polymer used) and drug load. The main polymer type was studied at three levels namely PLGA 5004A, PLGA 5010, and PCL. The low and the high level of each numeric factor were set to 0 and 25% (out of the total polymer weight) for Eudragit RS100 content and 40 to 160 mg in case of drug load; where Design-Expert software suggested 22 combinations with levels in between them.

On the other hand, drug EE% and *in vitro* drug release behavior from the prepared spherical beads (Q3h, Q6h and Q12h) were evaluated as dependent variables.

Polymeric solutions of different polymers were prepared as 150 mg total weight of polymers with different compositions in 1 ml dichloromethane as presented in Table 1. VP was added and thoroughly mixed in the solution. A 5 µl droplet of the solution was placed on the prepared superamphiphobic plates. Then, the plates were left for air drying for 24 h and later the beads were collected for further investigation (Rial-Hermida et al., 2014).

**Table 1.** Composition of VP-loaded polymeric beads adopting D-optimal design with their resultant dependent variables.

System Number	A: Polymer Type	B: Eudragit RS100 content (% w/w)^a^	C: Drug load (mg)	EE %± SD (% w/w)	Q 3hr ± SD (% w/w)	Q 6hr ± SD (% w/w)	Q 12hr ± SD (%w/w)
S1	PLGA 5004	0.00	109.40	98.02 ± 0.40	21.68 ± 11.40	31.72 ± 15.24	42.58 ± 15.10
S2		0.00	109.40	98.95 ± 0.14	17.50 ± 07.66	25.59 ± 09.60	37.53 ± 12.36
S3		10.41	40.00	93.41 ± 0.13	04.78 ± 01.11	04.79 ± 03.73	11.35 ± 02.62
S4		12.50	97.66	97.61 ± 0.03	20.18 ± 09.47	29.72 ± 12.76	40.27 ± 15.52
S5		14.57	160.00	98.11 ± 0.38	36.50 ± 12.04	49.36 ± 13.82	60.80 ± 12.13
S6		14.57	160.00	97.16 ± 0.42	46.64 ± 12.75	59.94 ± 15.11	73.07 ± 16.42
S7		21.88	40.00	92.94 ± 1.85	19.70 ± 03.84	19.27 ± 01.20	28.11 ± 00.56
S8		25.00	90.53	92.31 ± 1.79	42.15 ± 10.87	52.46 ± 10.09	64.72 ± 07.38
S9	PLGA 5010	0.00	40.00	96.68 ± 0.34	00.65 ± 00.58	02.73 ± 01.11	05.46 ± 05.59
S10		0.00	160.00	99.19 ± 0.03	32.14 ± 17.12	47.04 ± 21.13	65.35 ± 19.67
S11		0.00	160.00	99.30 ± 0.38	29.16 ± 05.70	44.27 ± 07.21	58.37 ± 05.89
S12		12.50	66.43	98.15 ± 0.40	06.65 ± 02.04	13.08 ± 03.55	21.98 ± 05.12
S13		12.50	130.10	98.88 ± 0.06	23.35 ± 00.52	35.93 ± 05.94	54.66 ± 09.53
S14		25.00	40.00	96.36 ± 0.32	22.35 ± 05.88	17.03 ± 02.26	27.39 ± 00.00
S15		25.00	160.00	97.29 ± 0.98	36.53 ± 05.86	55.62 ± 06.59	74.79 ± 05.97
S16	PCL	0.00	40.00	96.59 ± 0.31	39.25 ± 00.69	46.62 ± 00.73	61.08 ± 05.22
S17		0.00	40.00	96.30 ± 1.87	34.20 ± 00.39	37.56 ± 07.38	53.82 ± 08.97
S18		0.00	160.00	98.35 ± 0.06	52.79 ± 00.36	65.50 ± 00.80	75.36 ± 01.26
S19		0.00	160.00	98.94 ± 0.34	50.08 ± 04.94	61.01 ± 06.45	73.69 ± 06.89
S20		12.50	100.00	97.82 ± 0.64	47.79 ± 02.14	60.67 ± 01.68	69.65 ± 02.02
S21		23.05	59.82	97.50 ± 0.67	50.32 ± 05.24	59.42 ± 03.73	71.37 ± 03.54
S22		25.00	160.00	98.86 ± 0.08	72.69 ± 03.06	86.94 ± 05.99	95.90 ± 02.58

^a^Percent out of total polymer weight (150 mg).

All the 22 combinations were performed in duplicates and in a randomized form to satisfy the statistical requirements. A significance level of 5% was used as criterion to reject the null hypothesis. Statistical analyses were performed using analysis of variance (ANOVA) with Design-Expert^®^ software (version 7.0.0, Stat-Ease Inc., Minneapolis, MN) and the results were optimized.

### Characterization of the prepared VP-loaded polymeric beads

#### Encapsulation efficiency (EE%)

After complete drying and removal of the formulated beads, the superamphiphobic layer was divided into small parts, immersed into 100 ml of water and was magnetically stirred for 24 h to extract any residual amount of free un-encapsulated VP from the surface. On the other hand, the solidified beads were crushed and allowed to stir with water until complete release of all the encapsulated VP. Extracted drug in water was measured using a ultraviolet (UV) spectrophotometer (UV-1800; Shimadzu, Kyoto, Japan) at λ_max_ 278 nm. A blank superamphiphobic surface was scratched, magnetically stirred with water, and measured spectrophotometrically to assure the absence of any product that could interfere with the UV absorption of the drug. The percentage of the drug encapsulated was calculated from using Equation (1):

(1) $$\;\;\;\text{EE}\%=\frac{D_{e}}{D_{e}+D_{f}}\times \text{1}00\;\;\;$$

Where *D_e_* is the amount of VP encapsulated within the beads and *D_f_* is the amount of the free un-encapsulated drug.

#### *In vitro* drug release study

The release of VP from the formulated beads was monitored using the dissolution USP standard apparatus no. 1 (rotating basket) method (Varian, VK7000; Varian Inc., North Carolina, USA). The dissolution medium was chosen to be 300 ml 0.1 N hydrochloric acid (HCl;pH 1.2) for the first 2 h then, then 100 ml of 0.2 M tribasic sodium phosphate was added to raise the pH to 6.8 to represent the gastric and intestinal conditions taking into consideration that sink conditions were maintained all over the release period (24 h; <711> Dissolution, 2011).

Specified weight of VP-loaded beads was placed in the basket apparatus, immersed in the dissolution medium and incubated at 37 °C with speed of 100 rpm (Cohen et al., 1990). Three ml samples were withdrawn at predefined time points for 24 h duration (0.25, 0.5, 1, 1.5, 2, 3, 4, 6, 8, 10, 12, and 24 h, respectively) and were replaced with an equal volume of fresh medium. VP concentration in each sample was quantitatively determined using UV spectrophotometer at 278 nm. In addition, *in vitro* release of the market product; Isoptin^®^ immediate release tablets, was done for comparison purposes. VP release from Isoptin^®^ was evaluated from the tablet as a whole (80 mg) and upon dividing into one fourth (quarter) to assure the similarity in the release behavior. All the *in vitro* drug release studies were performed in duplicates.

According to Cohen et al. (1990), the required release duration (D) must be determined and the sustained release profile should be governed as follows: only 20–55% to dissolve in 0.25 D, between 45–75% in 0.5 D, and more than 75% within 1D. The first-time point is set to assure that there is no dose dumping, the second time point to characterize the pattern of the release profile while the last time point is to guarantee the complete release of the intended dose.

In our work, the required release duration was set to 12 h (D) and percent of VP released at different time points (Q3h, Q6h, and Q12h as 0.25D, 0.5 D, and 1 D, respectively) were used to compare the drug release profiles from different systems. All results were statistically analyzed via ANOVA by Design-Expert^®^ software.

#### Statistical optimization of the results

Statistical optimization of the results was done using Design Expert^®^ software to select the most desirable system from the prepared design where the criterion was set to the highest EE %, Q3 h in the range of (20–55%), Q6 h in the range of (45–75%), and more than 75% for Q12 h (Cohen et al., 1990). Further characterization was done on the optimized system.

### Characterization of the optimized system

#### Scanning electron microscopy (SEM)

The size, surface structure, and topography of the optimized formulated beads were observed using a SEM (Quanta 250 FEG; FEI company, Netherlands). The beads were mounted on metal grids, sprayed with gold (Emitech K550X sputter coater; Quorum Technologies, England) and photomicrographs were taken.

#### 4.2. Fourier transform infrared (FTIR) spectroscopy:

FTIR scanning was performed for VP, PCL, drug free PCL beads, and the optimized system. About 2–3 mg of each sample was ground, then mixed with 100 mg of potassium bromide, were compressed into thin discs using a hydrostatic press and finally were scanned with FTIR spectrophotometer (IR Affinity-1; Shimadzu, Kyoto, Japan) over wavelength range 4000–400 cm^−1^.

#### *In vivo* pharmacokinetic study

The optimized system; ‘OS’, was monitored for the *in vivo* performance study after filling into a hard gelatin capsule (‘OS’-capsule) and was compared to the immediate release market product Isoptin^®^. The capsule acted only as a reservoir to contain the formulated beads as it readily dissolves in the dissolution medium within few minutes and no difference was observed when the *in vitro* release of VP was re-investigated. Six male albino rabbits (body weight; 2–2.5 kg) were randomly allocated into two groups for a cross over study. Rabbits were housed according to National Institutes of Health guidelines and the study protocol was approved by Research Ethics Committee (REC) for experimental and clinical studies at Faculty of Pharmacy, Cairo University, Cairo, Egypt (PI (1343)). The rabbits were supplied by the Laboratory Animal Center at Faculty of Pharmacy, Cairo University, Egypt. Each rabbit was housed individually and allowed free access to food and water for the duration of the experiment.

A cross over study with one week washout period was applied where a single oral VP dose (equivalent to 10 mg/kg; Choi & Burm, 2005) of either Isoptin^®^ or the optimized system was administrated for each rabbit. Blood samples (3 ml) were withdrawn from the ear vein at predetermined time intervals (0.5, 1, 1.5, 2, 3, 4, 5, 6, 8, 12, and 24 h, respectively) into heparinized tubes and were centrifuged at 4000 rpm for 15 min (NF 815; Turkey). Finally the plasma was collected and stored frozen until liquid chromatography-mass spectrometry (LC/MS/MS) analysis.

#### Sample preparation and LC-MS/MS analysis

VP was determined in plasma samples using Triple Quadrupole LC/MS/MS Mass Spectrometer (AB Sciex Instruments, Framingham, MA). Hundred microliter of the internal standard stock solution (100 ng/ml of Torsemide) was added to the plasma sample (0.5 ml) and were vortexed. Extraction solvent (4 ml ethyl acetate) was added and the samples were vortexed together for 1 min and were later centrifuged for 10 min at 4000 rpm and 4 °C. The organic layer was separated and dried using vacuum concentrator (Eppendorf 5301; Hamburg, Germany). The dried residue was reconstituted with 100 µl of the mobile phase (Acetonitrile: 0.1% formic acid in water (80:20, v: v)) and finally, transferred to the autosampler vials where 10 µl was injected into the LC-MS/MS. The isocratic mobile phase was delivered at a flow rate of 1 ml/min. into the mass spectrometer’s electron spray ionization chamber.

A Shimadzu Prominence series LC system (Shimadzu Scientific Instruments, Kyoto, Japan) equipped with degasser (DGU-20A3) and pump (LC-20AD) with an autosampler (SIL-20A/HT) was used to inject 10 µl of the sample on a ZORBAX Eclipse plus C_18_ column (Agilent, Palo Alto, CA) 4.6 × 50 mm, 5 µm PS. The quantitation was achieved by MS/MS detection in the positive ion mode for both VP and IS using a MDS Sciex (Framingham, MA) API-4000 mass spectrometer, equipped with turbo ion spray interface at 450 °C. The ion spray voltage was set to 5500 V. Ions detection was performed in the multiple reactions monitoring mode (MRM), monitoring the transition of the m/z 455.27 precursor ion to the m/z 165.30 for VP and m/z 348.99 precursor ion to the m/z 263.90 for the internal standard. The analytical data were processed by Analyst^®^ Software Version 1.6 (AB Sciex).

##### Pharmacokinetic and statistical analysis

The obtained plasma concentration-time data was analyzed via non- compartmental pharmacokinetic model using Kinetica 2000 software (version 3.0, Monterey, CA) and the pharmacokinetic parameters of VP after oral administration of either Isoptin^®^ or the optimized system were estimated. Maximum plasma concentration (*C*_max._) and time to reach *C*_max._ (*T*_max._) were estimated directly from the plasma concentration-time profile. Elimination rate constant (k) was calculated from the terminal elimination line using the log-linear regression analysis and subsequently the half-life (*t*_1/2_) was determined as *t*_1/2_ = 0.693/K. The area under the curve (AUC_0-∞_) and the area under the first moment curve (AUMC_0-∞_) were calculated using the trapezoidal rule and consequently, the mean residence time (MRT) was determined by dividing (AUMC_0-∞_) by (AUC_0-∞_). Afterwards, the data were statistically analyzed to detect the significant difference between the values at *p* value < .05 via SPSS (SPSS^®^ Statistics software program, Version 17.0, International Business Machines Corp., Armonk, NY).

## Results and discussion

### Characterization of the prepared superamphiphobic substrates

Superamphiphobic substrates are substrates that possess both hydrophobic and oleophobic nature; i.e. show high contact angles (CA) for both water and oil liquids respectively (Manna et al., 2017). Due to the very low surface tension of the oil and the strong liquid-solid interaction, it’s usually hard to synthesize surface with low oil wettability (Deng et al., 2017). To design such surfaces, both surface morphology and surface composition should be strictly modified. Thus controlling surface roughness and surface free energy, by creating hybrid micro-nano hierarchical structure on the surface followed by its modification with low surface tension chemicals as fluoroalkylsilanes could be a successful approach for such surfaces (Ji et al., 2013; Peng & Deng, 2015).

**Figure 1.**
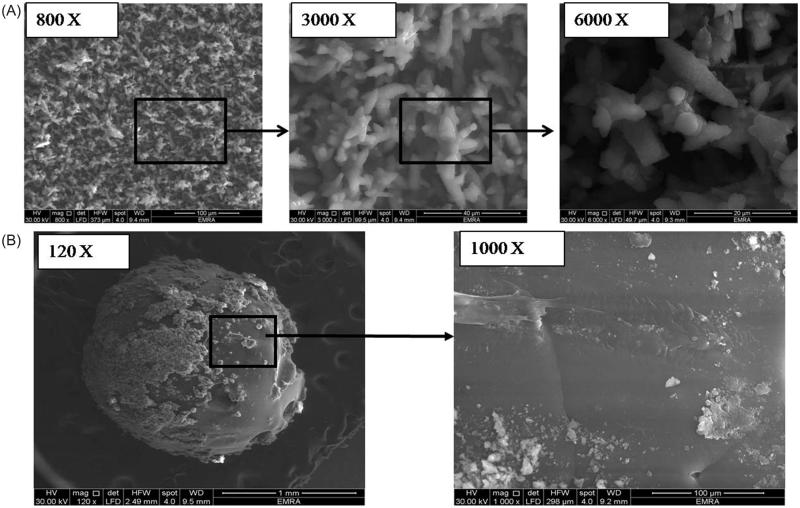
SEM images showing (A) the prepared superamphiphobic substrate with micro-length hierarchical structure at different magnification powers (800, 3000, and 6000 x, respectively) and (B) the optimized VP-loaded PCL based bead system (“OS”) with different magnification powers (120× and 1000×).

In our work, superamphiphobic surfaces were prepared using aluminum substrates. The hot ammonia solution was used to chemically etch the surface thus creating the required rough hierarchical structure. The ammonia solution generated (OH)− group that reacted with Al^3+^ ions to produce triangular clusters of aluminium hydroxide and aluminium hydroxide oxide (Peng & Deng, 2015). Then, the rough surface was immersed in alcoholic solution of PFDTS to derive the surface with Difluoromethane and trifluoromethane tails and thus, lower the surface free energy. PFDTS was chosen because of its chemical stability and its ability to interact with the surface under mild conditions (Deng et al., 2017). Figure 1(A) presents the SEM images of the substrate that show aggregates of triangular prisms in the micro-size range. Upon magnification, fine protrusions appear on the surface of such prisms that create the rough nano-micro hierarchical structure required to trap a large amount of air at the solid-liquid interface to prevent the penetration of oils and water into the grooves and control the surface wettability.

**Figure 2.**
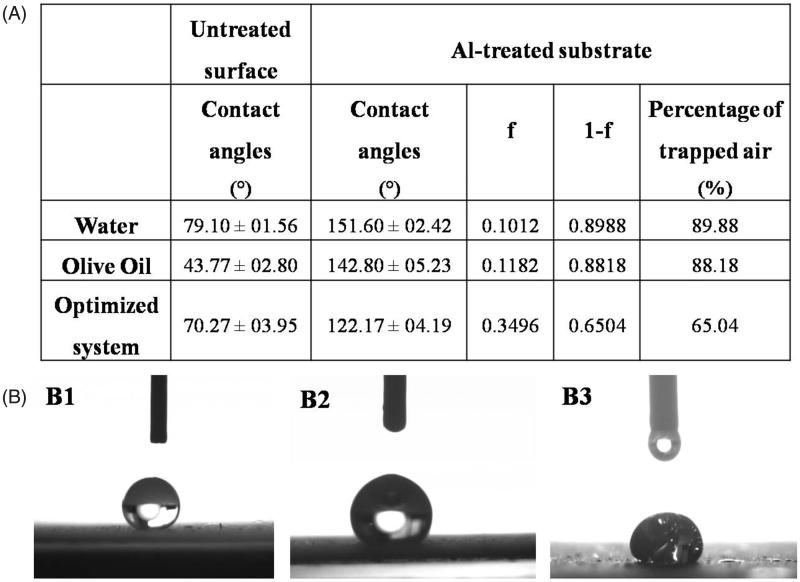
(A) Contact angles and percentages of the trapped air for different liquids on the superamphiphobic treated aluminum surface compared to the contact angles of the untreated surface; While (B) optical images of contact angles for water (B1), olive oil (B2), and the optimized VP-loaded PCL based solution (B3).

When a water droplet was dropped on the surface of the treated aluminum substrates, it showed a high CA (>150°) indicating the excellent superhydrophobicity of the surface (Figure 2(A,B)). Also, the surface showed a good oleophobicity (Sheen et al., 2008; Goto et al., 2011) as when a droplet of oil was dropped on the surface, a higher CA of (142.80° ± 05.23°) confirming the oleophobic property compared to the untreated surface.

The fraction of the air in contact with the liquid droplet and thus its percentage was deduced using Cassie and Baxter equation (Cassie & Baxter, 1944) as shown in Equation (2):

(2) $$\cos\theta_{*}=-1+f\left(\cos\theta +1\right)$$

where θ_*_ and *θ* are the CAs for the liquid droplet on the rough superamphiphobic- treated aluminum plates and the untreated plates, respectively; *f* is the fraction of solid treated surface in contact with the liquid droplet and accordingly, (1−f) is the fraction of the trapped air beneath the liquid droplet. Results presented in Figure 2(A) demonstrate that the large percentage of air trapped in the air pockets at the interface between the surface and the liquid which was approximately 90% is the main reason of preventing the liquid from spreading and wetting the surface (Peng & Deng, 2015).

In case of VP-PCL solution (the optimized system), the substrate still exhibits low wettability (>90°) and good oleophobicity (Goto et al., 2011) although the CA was slightly reduced to 122.17° and the fraction of the trapped air dropped to 65% as shown in Figure 2(A). This slight reduction in the CA could be attributed to external factors that affect the CA such as the gravity (Yuan & Lee, 2013). The high load of VP and PCL exerts an additional weight to the droplet and according to Newton’s law of universal gravitation (Newton, 1729), the gravitational force increases by increasing the mass of the body and thus, the high concentration of VP and PCL was translated into a higher gravitational force on the droplet which subsequently resulted in slight reduction in the CA (Figure 2(B)).

### Characterization of the formulated VP-loaded polymeric beads

The successfully prepared superamphiphobic substrates were used to prepare ultra-high VP loaded polymeric beads. PLGA and PCL; as hydrophobic polymers, were used to encapsulate and strictly control VP release from the formulated systems. Both polymers are widely used in drug formulations due to their biocompatibility and biodegradability. They show slow degradation rate that sustain the release of the encapsulated drug over a prolonged period of time (Acharya & Sahoo, 2011; Nordstrom, 2011; Alvarado et al., 2015). PCL has been broadly used in the formulation of the prolonged release systems specifically for water soluble drugs, possibly because it is known to exhibit slow rate of bioerosion (Song et al., 1997; Ramesh, 2009; Nordstrom, 2011). Eudragit RS 100 was also incorporated in the system formulation to modulate the high sustainability offered by the used hydrophobic polymers.

PCL and PLGA (namely PLGA 5004 A and 5010) were assessed either alone or in combination with Eudragit RS100 in the formulation of VP-loaded polymeric beads. Both PLGA types are of 50:50 lactide: glycolide molar ratio; however, they differ in that PLGA 5004 A has an inherent viscosity of 0.4 dl/g and an acidic terminal group whereas PLGA 5010; is of higher inherent viscosity (1 dl/g). The effect of the main polymer type used, Eudragit RS100 content, and drug load on the EE% and *in vitro* release was also studied and statistically optimized for further investigation of *in vivo* performance.

### Encapsulation efficiency (EE%)

Encapsulating hydrophilic drugs into multiparticulate systems always suffers from the problem of drug loss to the external phase (Yousry et al., 2016, 2017). In this study, beads formation and solidification took place on the previously prepared superamphiphobic substrates as discussed before. The absence of an external liquid phase during the preparation techniques resulted in ultrahigh EE% ranging from 92.31 to 99.30%w/w as shown in Table 1. Statistical analysis of the results via ANOVA was done using Design-Expert^®^ which suggested two factor interaction (FI) model as the model of choice for analysis where it showed significant effects of all the independent variables analyzed as well as a significant two-factor interaction between the main polymer type and Eudragit RS100 content.

### Effect of main polymer type

**Figure 3.**
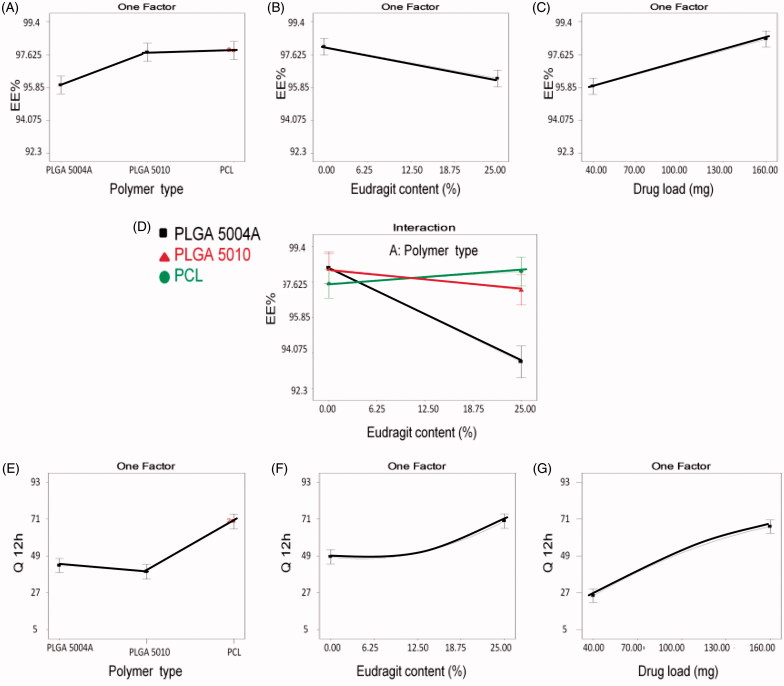
Line plot presenting the effect of the variables on: entrapment efficiency (EE%): Effect of (A) main polymer type, (B) Eudragit RS100 content, (C) drug load, and (D) two factor interaction between polymer type and Eudragit RS100 content. *In-vitro* release (Q12hr): Effect of (E) main polymer type, (F) Eudragit RS100 content, and (G) drug load.

The main polymer type significantly (*p <* .05) affected the EE% where PLGA 5004 A among other polymers showed significantly lower EE% (Figure 3(A)), whereas; there was no significant difference between PCL and PLGA 5010. This effect could only be understood in light of the significant interaction (*p <* .05) between the polymer type and Eudragit RS100 content (Figure 3(D)) as discussed under the following section.

### Effect of Eudragit RS100 content

Figure 3(B) shows that Eudragit RS100 addition in different concentrations significantly reduced EE%, whereas Figure 3(D) showing the interaction between the main polymer type and Eudragit RS100 content explains the pattern of such effect. At 0% Eudragit RS100, there was no significant difference in the EE% between the three main polymers investigated. Upon increasing Eudragit RS100 content, the EE% was significantly reduced in case of PLGA 5004 A and slightly changed in case of both PLGA 5010 and PCL. This could be related to the structure of the used polymer; PLGA 5010 and PCL are neutral compounds with no charge (Hoffart et al., 2002), PLGA 5004A- being acid terminated- is negatively charged whereas Eudragit RS100 is positively charged due to the presence of quaternary ammonium group. When Eudragit RS100 is added to PLGA 5004A, the positively charged ammonium group of Eudragit RS100 could electrostatically interact with the negatively charged carboxylic group of PLGA 5004 A polymer replacing some of the encapsulated drug within the polymer matrix and expelling it outside the beads resulting in significant reduction in EE%. This effect is not prominent with other polymers (PLGA 5010 and PCL) as they are neutral compounds.

### Effect of drug load

Increasing drug load showed a significant effect (*p <* .05) on the EE % as shown in Figure 3(C) where VP EE% was increased upon increasing the drug load in the formulated systems. Similar results were obtained while formulating different multiparticulate systems (Patel et al., 2006; Piyakulawat et al,. 2007; Mobarak et al., 2014; Gupta et al., 2016). In this study, VP and the polymers were completely soluble in methylene chloride and fast solidification of the beads occurred due to the high volatility of the solvent. It could be possible that the un-encapsulated VP is lost by diffusion from the outer layer of the solvent in contact with the superamphiphobic surface where beads solidification occurs before any further migration of VP takes place from the inner layers of the viscous solution to the external surface. Thus, the un-encapsulated diffused amount of VP from the outer layer was almost the same in all the prepared systems, therefore, upon increasing the drug load, higher EE% was obtained.

### *In vitro* drug release

The *in vitro* drug release from the prepared systems was studied to evaluate and compare the beads behavior regarding drug release and to predict the *in vivo* behavior upon administration. All the prepared systems showed controlled VP release within the observed 24 h. A very slow VP release was observed with some systems where the quantity released after 24 h was only less than 10% of the encapsulated drug.

Three-time points namely; Q3h, Q6h, and Q12h were statistically analyzed via ANOVA. Design-Expert^®^ suggested quadratic model as the model of choice for analysis and it shows significant effects of all the independent variables analyzed with the same pattern on the three dependent variables measured.

### Effect of main polymer type

Drug release rate depends mainly on the polymers used in the formulation where the more hydrophobic polymers (Jiao et al., 2002) and the higher molecular weight polymers (Balmert et al., 2015) hinder drug release from the system.

The main polymer type had a significant effect (*p <* .05) on the *in vitro* release, where PCL resulted in a significant higher VP release compared to PLGAs (Figure 3(E)) whereas PLGA 5004 A showed slightly higher release compared to PLGA 5010. This could be explained in terms of polymer structure which affects polymer degradation and drug diffusion rate.

PLGA degrades by hydrolysis of its ester linkages through bulk erosion in four consecutive steps namely; hydration; where water penetrates into the amorphous region disrupting the van der Waals forces and hydrogen bonds followed by initial degradation via cleavage of the covalent bonds. The resulted carboxylic end groups of the degradation products (lactic and glycolic acids) autocatalyze further constant degradation of the back bone covalent bonds which results in loss of integrity. The carboxylic termination of PLGA 5004 A enhances its degradation as it renders the molecule more polar than PLGA 5010 promoting more water penetration inside the polymer molecule which increases its degradation rate. This carboxylic group also autocatalyzes the back bone degradation process resulting in an overall higher polymer degradation and drug release compared to PLGA 5010 (Gentile et al., 2014).

However, this difference between the two PLGAs degradation rate and its effect on VP release was not emphasized because once the degradation process started, the anionic carboxylic - ended fragments produced from both polymers in addition to the acid terminated group of PLGA 5004 A would interact with the positively charged VP (pKa = 8.6; being protonated in the acidic release medium) retarding its diffusion and resulting in an overall slow release pattern of the PLGAs (Khamanga & Walker, 2011).

On the other hand, PCL hydrolytic degradation occurs via surface or bulk degradation pathways. Surface degradation involves hydrolytic cleavage of the polymer backbone on the surface and this takes place when the water can’t find its way to the bulk polymer resulting in the diffusion of the oligomers and monomers to the surroundings and subsequently thinning of the surface layer of the polymer and cracks formation. Whereas bulk degradation occurs when water penetrates the entire polymer bulk and hydrolyzes the entire polymer matrix. This process of polymer degradation is enhanced in the less crystalline PCL structure, whereas, PCL crystallinity is reduced upon increasing its M. Wt. Thus, the used high M. Wt. of PCL renders it more susceptible to hydrolytic degradation and crack formation which could explain the overall faster release observed with VP relative to the other PLGA polymers (Sinha et al., 2004; Woodruff & Hutmacher, 2010).

### Effect of Eudragit RS100 content

Eudragit RS100 content showed a significant positive effect (*p <* .05) on the percent VP release from the formulated beads. Eudragit RS100 is widely used to control drug release. Thus, the significant positive effect (Figure 3(F)) resulted from the addition of Eudragit RS100 on the release is uncommon (Fürst, 2009; Sonje & Chandra, 2013). This effect could be explained based on the chemical and physical structure of Eudragit RS100 as it is a copolymer of Ethyl acrylate, methyl methacrylate, and a low content of methacrylic acid with quaternary ammonium groups that render the polymer permeable with a pH independent swelling behavior (Sonje & Chandra, 2013).

Upon replacing part of the main hydrophobic impermeable polymers (PLGAs and PCL) with Eudragit RS100 in different concentrations, the permeability of the beads to the dissolution media increases. The dissolution medium that finds its way inside the beads can dissolve the encapsulated VP and enhances its release to the external release medium. In addition, the dissolution medium enhances the hydrolytic cleavage and degradation of the polymers rendering them more permeable to drug diffusion. Tao et al. (2009) found similar results when they incorporated Carbopol in their acylclovir- loaded, ethylcellulose- based microspheres. They explained that based on the water solubility of Carbopol that created hydrophilic passages inside the microspheres which enhanced the rate of drug diffusion out.

### Effect of drug load

Drug load showed a significant effect (*p* < .05) as shown in Figure 3(G) where VP release was enhanced by increasing its load inside the beads. This effect was expected due to the high drug concentration gradient between the beads and the external dissolution medium. This concentration gradient drives the drug to diffuse more to the lower drug concentration phase and increases the release. Also, the hydrophilic VP which is embedded in the formulated beads acts like hydrophilic pores that create an exit pathway to the remaining molecules of the drug. When drug load increases, the polymer content of the beads will decrease resulting in the formation of more exit hydrophilic channels that boost VP release (Prasertmanakit et al., 2009). This effect is common with different multiparticulate systems (Akbu & Durmaz, 1994; Bayomi & Mesnad, 1998; Mainardes & Evangelista, 2005).

### Statistical optimization of the data

Statistical optimization of the effect of polymer type, Eudragit RS100 content and drug load on the EE %, Q3 h, Q6 h, and Q 2 h of VP-loaded polymeric beads was done using Design Expert 7.0.0 software. The optimization conditions were set to the highest EE %, Q3h (20–55%), Q6h (45–75%), and Q12h (>75%).

**Figure 4.**
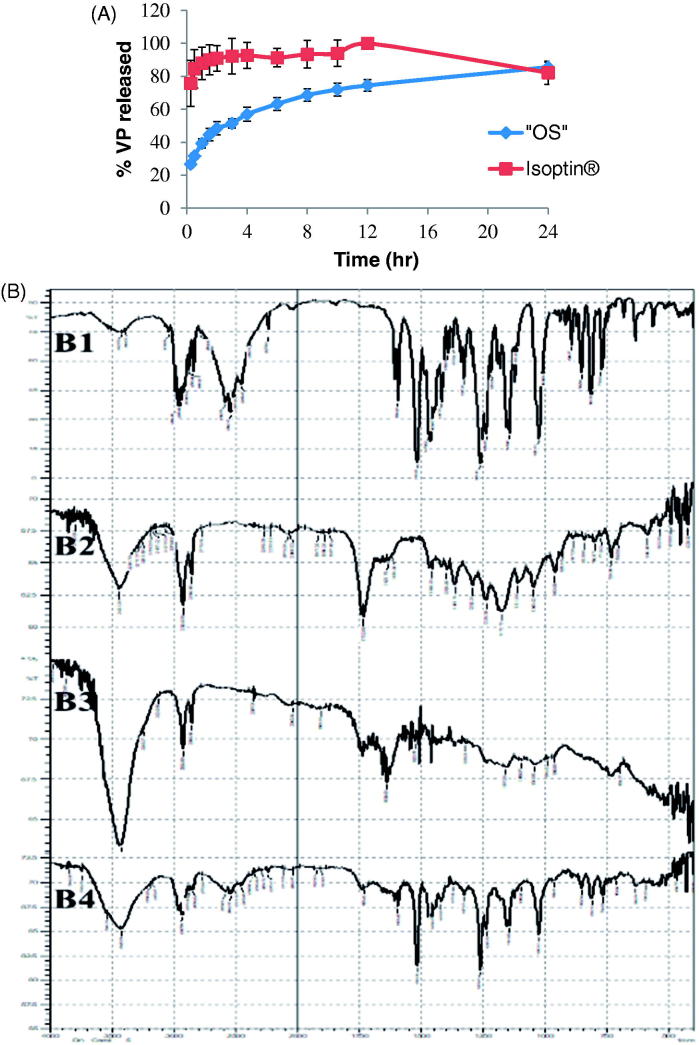
(A) Release profiles of VP from the optimized system (“OS”) compared to the market product (Isoptin^®^); while (B) shows the FTIR spectrum of pure VP (B1), PCL (B2), drug free PCL beads (B3), and optimized VP-loaded PCL beads (B4).

Optimization results revealed that the system composed of 150 mg PCL (1.5% w/v) without Eudragit RS100 and 160 mg VP is the system of choice ‘OS’ with desirability factor 0.916. This optimized system is the same as the previously formulated system S18 and S19 (duplicate formulations; Table 1). The release profile of the optimized system ‘OS’ in comparison to the market product Isoptin^®^ is shown in Figure 4(A) revealed that the release of VP from the optimized system exhibited more controlled behavior than the immediate release market product. Kinetic analysis of VP release from the optimized system showed that the release profile of ‘OS’ followed Higuchi diffusion model with *r*^2^ value of 0.98.

### Characterization of the optimized systems

#### Scanning electron microscope

SEM image (Figure 1(B)) of the formulated VP-loaded PCL based system (‘OS’) showed smooth spherical shape with average size of 1.71 ± 0.10 mm. Some clusters of the drug appear attached to the surface, these represent the surface drug that burst initially within the first 15 minutes in the *in vitro* release study.

#### FTIR spectroscopy

VP spectrum (Figure 4(B1)) shows C-H stretching peaks of methylene and methoxy groups at (2954 – 2839 cm^−1^), a sharp characteristic peak of –C≡N at 2237 cm^−1,^ C-H stretching of the benzene ring at 1593, 1516, and 1473 cm^−1^ and a strong C-O stretching vibrations of the aromatic ethers at 1261 cm^−1^ (Saleem et al., 2012; Tekade et al. 2014). Also, the FTIR spectrum of PCL in Figure 4(B2) shows C = O stretching at 1732 cm^−1^, OC- O stretching at 1168 cm^−1^, asymmetric CH_2_ group at 2926 cm^−1^, and symmetric CH_2_ group at 2864 cm^−1^ (Elzubair et al., 2006). Figure 4(B3) retains all the characteristic peaks of PCL and Figure 4(B4) retains the characteristic peaks of VP and PCL without shifting assuring the absence of any chemical interactions in the beads formulation process. Attenuation of some characteristic peaks occurs due to drug dilution during formulation.

#### *In vivo* pharmacokinetic study

The *in vivo* behavior of VP-loaded PCL beads (‘OS’) filled capsules was assessed and compared to Isoptin^®^ by monitoring VP plasma level for 24 h post-oral administration to six male albino rabbits in a cross over design. Kunta et al., (2004) found that the rabbits model is more suitable than dogs, rats, and mice in monitoring VP clinical pharmacokinetics and drug interactions which take place in rabbits in patterns similar to those of human.

**Figure 5.**
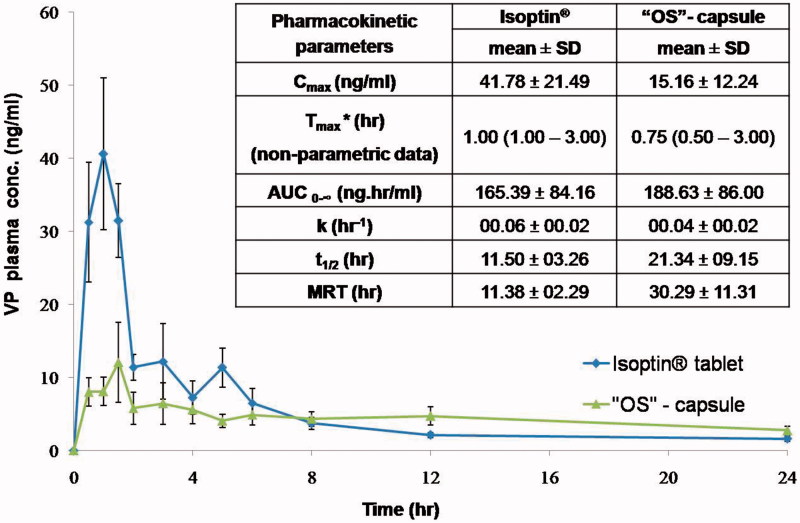
Mean VP plasma concentration- time curve and mean pharmacokinetic parameters of VP following oral administration of Isoptin^®^ and VP-loaded PCL beads (“OS”) filled capsules to six albino rabbits.

Plasma concentration-time curves for the optimized system ‘OS’-filled capsules (‘OS’ -capsule) and the market product (Isoptin^®^) are represented in Figure 5. In the first hours post administration, the plasma profile of Isoptin^®^ showed a remarkably (*p* < .05) significant higher plasma concentration compared to ‘OS’-capsule followed by a fast decline in the subsequent elimination phase. On the other hand, the formulated ‘OS’exhibited a slowly declining elimination curve that maintained VP plasma concentration higher for a prolonged time.

The average pharmacokinetic parameters (*n* = 6) for each system were determined by non-compartmental analysis model and summarized in Figure 5. Upon statistical analysis; *T*_max._, K, *t*_1/2_ and AUC_0-∞_ didn’t show any significant difference (*p* > .05) between the optimized system (‘OS’ -capsule) and the market product (Isoptin^®^). The insignificantly difference in the AUC_0-∞_ values of both systems indicates the comparable VP bioavailability upon sustained release formulation although it suffers from an extensive first-pass effect (Marvola et al., 1985).

It is clear from Figure 5 that the *C*_max._ is significantly different (*p* < .05) in both systems. The higher *C*_max._ (41.78 ± 21.49  ng/ml) in Isoptin^®^ compared to 15.16 ± 12.24 ng/ml in case of ‘OS’ -capsule was anticipated due to the rapid and immediate release of VP that results in a high plasma concentration.

Although of the insignificantly lower *T*_max._, Figure 5 demonstrates that the MRT of VP-loaded PCL beads (“OS”-capsule) is 30.29 ± 11.31h which is 2.7 folds compared to that of Isoptin^®^ (11.38 ± 2.29 h). A significant higher (*p* < .05) MRT from the optimized formulated system (‘OS’ -capsule) suggests a longer residence of VP molecules in the body assuring that the optimized formulated system offered a sustained release behavior *in vivo* as what was formerly observed *in vitro*.

Similar results were found by other researchers during preparation of controlled release systems where Ravi et al. (2014) formulated Lopinavir- loaded nanoparticles with 1.3 folds significantly higher MRT than free lopinavir although exhibiting the same *T*_max._ Also, Sreenivasa Rao et al. (2001) observed similar results while loading rifampicin into ethyl cellulose coated nonpareil beads.

## Conclusion

In the present study, superamphiphobic substrates were prepared successfully by etching aluminum plates followed by PFDTS treatment. Ultrahigh VP-loaded polymeric multiparticulate systems were formulated using the prepared superamphiphobic substrates with an extremely high encapsulation efficiency exceeding 92%; thus, it could be advantageous over other conventional encapsulation techniques. The effect of the main polymer type, Eudragit RS100 content and drug load on EE% and *in vitro* release of VP was investigated. The optimized system composed of 150 mg PCL (1.5% w/v) and drug load of 160 mg VP showed EE% of 98.72% w/w and 52.71%, 64.35 and 75.39% for Q3 h, Q6 h and Q12 h, respectively. *In vivo* pharmacokinetic study was carried out in comparison to the market product (Isoptin^®^) where it showed a comparable VP bioavailability together with 2.7 folds higher mean residence time (30.29 ± 11.31 h) allowing once daily administration instead of three times per day in case of Isoptin^®^ satisfying higher patient compliance.
